# Mortality Statistics

**Published:** 1882-01

**Authors:** 


### MORTALITY STATISTICS.

From statistics given up to the 15th of Oct., 1881, the following facts appear.

The six healthiest cities in the United States are Omaha, San Francisco, Worcester, Portland, Indianapolis, and New Haven. The death rate, per thousand, annually, in the order named is as follows, viz.: Omaha 13.7, San Francisco 16.1, Worcester 16.1, Portland 17, Indianapolis 18.1, New Haven 19.1

The six unhealthiest, according to the same showing, are Wilmington, Del., 43.2, Savannah 42.5, Buffalo 39.6, Atlanta 37.1, Nashville 36, Memphis 35.7.

The six unhealthiest cities in the world were, according to this report, Alexandria, Egypt, 48.8, St. Petersburg, Rus., 43.9, Wilmington, Del., Savannah, Buffalo, and Atlanta. Though the ratio of mortality in any given place will vary, in different seasons and years, yet statistics of this sort are interesting and instructive. Sanitary science is a subject in which all classes of people ought to take an interest, and seek information, and at once put into practical exercise all such information.

A large per cent. of deaths in any city and district from which reports are made is from preventable diseases, or at least from those that can be largely modified and controlled and that, too, by the right use of knowledge which is possessed by, or is within easy reach of any one of common intelligence. Everybody knows that infected air and water are adverse to health, and yet how little effort is made to secure either.

Almost anybody knows that a large proportion of the food in common use is, to say the least innutritious, and much of it positively hurtful, and some of it actually poisonous. The extent to which the adulteration and vitiation of the common and standard articles of food is carried is truly alarming.

Pure air, pure water, pure nutritious food, personal cleanliness, the avoidance of all excesses, both physical and mental, would renovate the race and put the average longevity above fifty years. Try it and see.

